# The prognostic value of D-dimer levels in endometrial cancer patients treated with intensity-modulated radiation therapy

**DOI:** 10.18632/oncotarget.15805

**Published:** 2017-02-28

**Authors:** Xiaojing Yang, Hanru Ren, Yi Sun, Lihua Zhang, Xinmiao Yang, Hongling Li, Yuhui Shao, Jie Fu

**Affiliations:** ^1^ Department of Radiation Oncology, Shanghai Jiao Tong University Affiliated Sixth People's Hospital, Shanghai, China; ^2^ Department of Orthopaedics, Shanghai Pudong Hospital, Fudan University, Pudong Medical Center, Shanghai, China

**Keywords:** D-dimer, endometrial cancer, prognosis, intensity-modulated radiation therapy

## Abstract

Explain the important role of plasma D-dimer in cancers. Plasma D-dimer is increased in various tumors. However, the predictive value of plasma D-dimer is unclear. This study is aimed to evaluate the prognostic value of the D-dimer level in patients managed with intensity-modulated radiation for endometrial cancer. The D-dimer levels of forty patients with endometrial cancer were assessed before (D1) and after (D2) intensity-modulated radiation therapy (IMRT), respectively. The D-dimer level changes (ΔD) were defined as D2 minus D1. Cox regression and log-rank tests were used to evaluate the D-dimer levels in relation to progression free survival (PFS) and overall survival (OS). The OS and PFS of patients with a low D1 were significantly longer than those with a high D1 (*P*< 0.001, *P*< 0.001). We saw the similar correlation between D2, PFS and OS (*P*< 0.001, *P*< 0.001). Multivariate survival analyses showed that D-dimer was independently associated with OS and PFS in patients with endometrial cancer. The ΔD level was not related to the OS and PFS in endometrial cancer patients. The levels of D-dimer may be considered as an important predictor of PFS and OS in endometrial cancer patients treated with IMRT.

## INTRODUCTION

Endometrial cancer is a common gynaecological malignancies [1]. Although hysterectomy is the first choice of treatment for endometrial cancer, postoperative pelvic radiotherapy and intensity-modulated radiation therapy (IMRT) is becoming popular and improve the clinical outcome [2]. However, the clinical outcome is not remarkable improved after IMRT in most patients. Biomarkers may enable the identification of endometrial cancer patients who are likely to benefit from IMRT [3]. Thus, development of simple and accurate prediction makers for the prognosis of endometrial cancer patients treated with IMRT after hysterectomy.

Tumor cells activate the coagulation pathway in endothelial cells, resulting in the secretion of procoagulants and a secondary increase of fibrinolysis and fibrin degradation product D-dimer [4, 5]. D-dimer stimulates the growth of malignancies via promoting tumor cell proliferation, adhesion and angiogenesis [6]. Several studies report that the level of D-dimer is increased in breast [7], gastric [8], colorectal [9], lung [10], and nasopharyngeal carcinomas [11], and is correlated with a poor prognosis and decreased response to treatment. However, the relation of D-dimer levels to the survival of patients with endometrial cancer has not been reported.

In this study, the plasma D-dimer levels were measured in patients treated with hysterectomy and postoperational IMRT for endometrial cancer. The prediction value of D-dimer in relation to progression free survival (PFS) and overall survival (OS) was also investigated.

## RESULTS

### Clinicopathological features

Forty patients with complete clinical data were included in this study. The median age was 56 years (range, 32-76). Clinicopathologic characteristics were shown in Table 1. 77.5% of the patients had a body mass index (BMI) index ≥ 25. Five percent of people had a history of smoking. The percent of patients had hypertensive and diabetic mellitus diseases were 72.5% and 67.5%, respectively. More than half of the patients had a family history of tumor diseases. 67.5% (27/40) of the patients had a Karnofsky Performance Scale (KPS) score ≥ 80. The plasma D-dimer level before (D1) and after IMRT (D2) were collected in 40 patients with endometrial cancer. The median D1, D2, and the difference in D-dimer levels (ΔD) were 2.42 (range 0.14–17.66) mg/L, 2.97 (range 0.13–21.37) mg/L, and 1.51 (range-1.36–9.0) mg/L, respectively. Based on the ROC analyses, the best cut-off value of D1, D2, and ΔD were 1.82mg/L, 1.90 mg/L, and 0.77 mg/L, respectively.

**Table 1:** Clinicopathologic characteristics

Characteristics	Median (25th–75thpercentile) or no. (%)
Median age, years (range)	56 (32-76)
BMI (kg/m2)	
< 25	9(22.5%)
≥ 25	31(77.5%)
Smoking	
No	38(95.0%)
Yes	2(5.0%)
Hypertensive	
No	11(27.5%)
Yes	29(72.5%)
Diabetic Mellitus	
No	13(32.5%)
Yes	27(67.5%)
Family History of Cancers	
No	17(42.5%)
Yes	23(57.5%)
KPS	
<80	13(32.5%)
≥80	27(67.5%)
Test	
D1(mg/L)	2.42 (0.14–17.66)
D2(mg/L)	2.97 (0.13–21.37)
ΔD (mg/L)	1.51 (-1.36–9.08)

Abbreviations: Body mass index: BMI; Karnofsky Performance Scale: KPS; D-dimer level before radition: D1; D-dimer level after radition: D2; Change in D-dimer level (D2 minus D1): ΔD.

### Univariate and multivariate analyses of prognostic factors

The data of the univariable analyses were shown in Table 2. We found that International Federation of Gynecology and Obstetrics (FIGO) stage (*P*< 0.001, *P*< 0.001), histologic grade (*P*= 0.001, *P*< 0.001), depth of myometrial invasion (MI) (*P*< 0.001, *P*< 0.001), lymph node metastasis (*P*= 0.001, *P*< 0.001), D1 (*P*< 0.001, *P* <0.001), and D2 (*P* <0.001, *P* <0.001) were significantly associated with OS and PFS. Multivariate analysis using the Cox proportional hazards model demonstrated that FIGO stage (hazard ratio (HR) = 33.338, 95 % confidence interval (CI): 0.259-49.805, *P*= 0.001; HR= 23.293, 95 % CI: 0.374-541.274, *P*< 0.001, respectively), D1 (HR= 33.530, 95 % CI: 2.319-42.066, *P*< 0.001; HR= 13.978, 95 % CI: 1.080-27.588, *P*= 0.001, respectively) as well as D2 (HR= 8.121, 95 % CI: -10.938-31.372, *P*= 0.031; HR= 9.924, 95 % CI: -4.580-29.401, *P*= 0.036, respectively) were independent prognostic indicator of OS and PFS (Table 3).

**Table 2:** Univariate analysis of factors associated with OS and PFS

	Cases (n)	OS		PFS	
		percent	*P*-value	percent	*P*-value
Age(years)					
<55	19	42.1%	0.185	42.1%	0.286
≥55	21	23.8%		28.6%	
Smoking					
No	38	52.6%	0.382	42.1%	0.205
Yes	2	50%		0%	
Hypertensive					
No	11	63.6%	0.211	54.5%	0.179
Yes	29	55.2%		41.4%	
Diabetic Mellitus					
No	13	61.5%	0.105	53.8%	0.146
Yes	27	37.0%		33.3%	
Family History of Cancers					
No	17	64.7%	0.091	70.6%	0.076
Yes	23	34.8%		30.45	
FIGO stage					
II	18	66.7%	<0.001	72.2%	<0.001
III	10	10.0%		10.0%	
IV	12	0.0%		0.0%	
Histologic grade					
G1	20	60.0%	0.001	65.0%	<0.001
G2	11	8.3%		8.3%	
G3	9	0.0%		0.0%	
Depth of MI					
< 50%	25	52.0%	<0.001	56.0%	<0.001
≥ 50%	15	0.0%		0.0%	
Lymph node metastasis					
No	22	54.5%	0.001	59.1%	<0.001
Yes	18	5.6%		5.6%	
BMI (kg/m2)					
< 25	9	33.3%	0.624	44.4%	0.381
≥ 25	31	32.3%		32.3%	
ER					
Negative	19	31.2%	0.587	21.1%	0.076
Positive	21	33.3%		47.6%	
PR					
Negative	17	47.1%	0.089	52.9%	0.044
Positive	23	21.7%		21.7%	
D1					
low	23	56.5%	<0.001	60.7%	<0.001
high	17	0.0%		0.0%	
D2					
low	17	76.5%	<0.001	82.4%	<0.001
high	23	0.0%		0.0%	
ΔD					
low	27	40.7%	0.105	44.4%	0.071
high	13	15.4%		15.4%	
KPS					
<80	13	30.8%	0.584	30.8%	0.491
≥80	27	33.3%		37.0%	

Abbreviations: D-dimer level before radition: D1; D-dimer level after radition: D2; Change in D-dimer level (D2 minus D1): ΔD; International Federation of Gynecology and Obstetrics: FIGO; G1: Well; G2: Moderate; G3: Poor; Myometrial invasion: MI; Body mass index: BMI; Estrogen receptor: ER; Progesterone receptor: PR; Progression-free survival: PFS; Overall survival: OS; Karnofsky Performance Scale: KPS.

**Table 3:** Multivariate analysis of factors associated with OS and PFS

	OS			PFS		
	95% CI	HR	*P*	95% CI	HR	*P*
Age	-11.307-3.428	0.3	0.104	-14.456-1.945	0.2	0.045
FIGO stage	0.259-49.805	33.3	0.001	0.374-541.274	23. 3	0.000
Histologic grade	-20.704-7.627	0.6	0.049	-16.556-4.512	0.4	0.192
Depth of MI	-16.325-13.405	0.8	0.807	-9.019-13.257	0. 9	0.911
Lymph node metastasis	-34.856-3.880	0.3	0.240	-25.089-4.635	0.4	0.486
BMI	-4.950-19.910	1.8	0.352	-1.512-17.916	1.8	0.358
D1	2.319-42.066	33.5	0.000	1.080-27.588	14.0	0.001
D2	-10.938-31.372	8.1	0.031	-4.580-29.401	9.9	0.036
ΔD	-19.473-2.738	0.3	0.069	-18.178-1.014	0.3	0.029
KPS	-1.599-21.148	1.6	0.079	-2.464-15.843	1.8	0.329

Abbreviations: D-dimer level before radition: D1; D-dimer level after radition: D2; Change in D-dimer level (D2 minus D1): ΔD; International Federation of Gynecology and Obstetrics: FIGO; Myometrial invasion: MI; Body mass index: BMI; Progression-free survival: PFS; Overall survival: OS; Karnofsky Performance Scale: KPS

### D-dimer as a prognostic factor of survival

**Figure 1:**
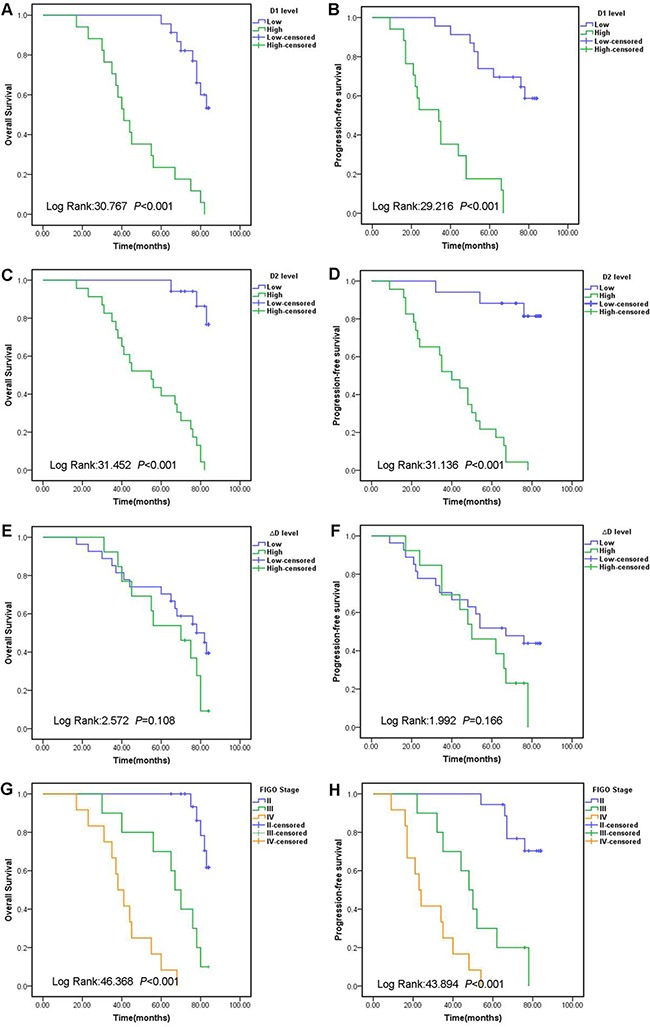
Kaplan-Meier survival curves for OS and PFS for endometrial cancer patients according to D1 levels (A, B), D2 levels (C, D), ΔD levels (E, F), and different FIGO stage (G, H) Abbreviations: Overall survival: OS; Progression free survival: PFS; D-dimer level before radition: D1; D-dimer level after radition: D2; Change in D-dimer level (D2 minus D1): ΔD; International Federation of Gynecology and Obstetrics: FIGO.

Kaplan–Meier analysis was performed to the levels of D-dimer on D1, D2 in relation to patients’ survivals. Based on the cut-off values described above, we divided patients into subgroups with lower level of D-dimer or higher level of D-dimer. The median OS and PFS times of all patients were 48 and 32 months, respectively. The Kaplan–Meier survival curves indicated that the OS and PFS of patients with a higher level of D-dimer on D1 were significantly shorter than those with a lower level on D1 (Figure 1A, B, *P*< 0.001, *P*< 0.001, respectively). Similar correlation was found between OS and PFS and D-dimer on D2 (Figure 1C, D, *P*< 0.001, *P*< 0.001, respectively). There was no differences in OS and PFS between patients with a higher ΔD and lower ΔD (Figure 1E, 1F, *P*= 0.108, *P*= 0.166, respectively). As shown in Figure 1G, 1H, endometrial cancer patients in early stage had better prognosis.

**Figure 2:**
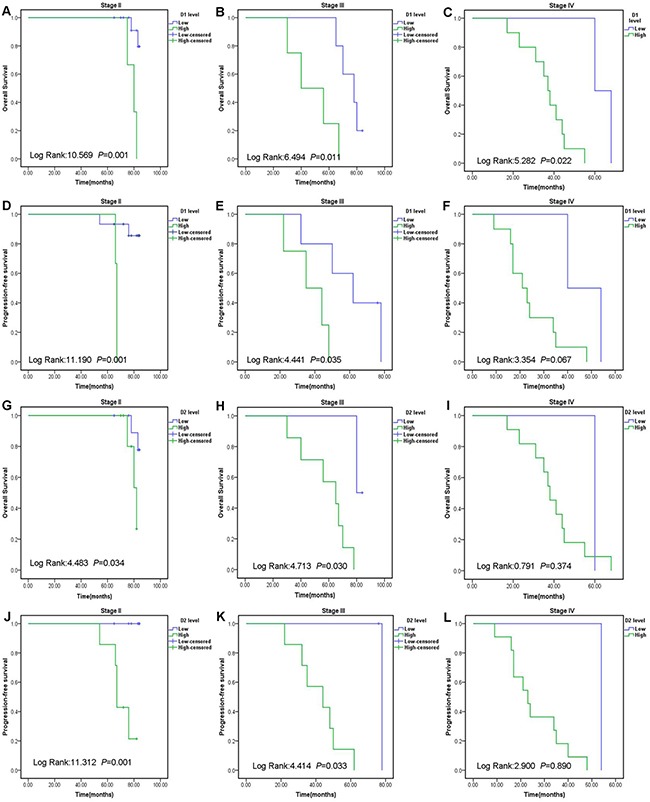
Prognostic significance of D-dimer in endometrial cancer patients according to the different FIGO stages Patients were divided into low and high groups based on the D1 or D2 levels cut-off values. The OS and PFS rate were calculated by the Kaplan–Meier method and analyzed by the log-rank test. Abbreviations: International Federation of Gynecology and Obstetrics: FIGO. D-dimer level before radition: D1; D-dimer level after radition: D2; Overall survival: OS; Progression-free survival: PFS.

Moreover, to detect the prognostic value of D-dimer in endometrial cancer patients, the premonitory affection of the levels of D-dimer on D1 and D2 were analyzed in the subgroup stratified by the FIGO stage II–IV. The mean concentrations of D-dimer on D1 in patients with FIGO stage II, III, IV stages were 0.33±0.54mg/L, 1.76±1.90mg/L, 3.81±4.33mg/L, respectively. The mean concentrations of D-dimer on D2 in patients with FIGO stage II, III, IV stages were 1.89±2.02mg/L, 3.12±4.61mg/L, 6.71±6.04mg/L, respectively. Patients with a higher level of D-dimer on D1 or D2 had remarkably shorter OS and PFS than the patients with a lower level of D-dimer on D1 or D2 level in patients with the stage II and III subgroups (Figure 2). As shown in Table 4, the levels of D-dimer on D1 and D2 were closely related to FIGO stage (*P*= 0.001; *P*< 0.001), histologic grade (*P*= 0.009; *P*< 0.001), depth of MI (*P*< 0.001; *P*< 0.001), lymph node metastasis (*P*= 0.001; *P*< 0.001), PFS (*P*< 0.001; *P*< 0.001) and OS (*P*< 0.001; *P*< 0.001). However, there was no association between the levels of D-dimer on D1 and D2 and other prognostic factors such as age, BMI, ER, PR expression. ΔD levels had no correlation to the above factors.

**Table 4:** Relationship between D-dimer level and the clinicopathological variables

	Cases (n)	D1		*P*	D2		*P*	ΔD		*P*
		Low	High		Low	High		Low	High	
Age(years)										
<55	19	11	8	0.607	9	10	0.393	12	7	0.413
≥55	21	12	9		8	13		15	6	
FIGO stage										
II	18	15	3	0.001	15	3	<0.001	15	3	0.064
III	10	6	4		2	8		4	6	
IV	12	2	10		0	12		8	4	
Histologic grade										
G1	20	16	4	0.009	15	5	<0.001	16	4	0.145
G2	11	5	6		2	9		5	6	
G3	9	2	7		0	9		6	3	
Depth of MI										
< 50%	25	20	5	<0.001	17	8	<0.001	17	8	0.599
≥ 50%	15	3	12		0	15		10	5	
Lymph node metastasis										
No	22	18	4	0.001	16	6	<0.001	16	6	0.329
Yes	18	5	13		1	17		11	7	
BMI (kg/m2)										
< 25	9	5	4	0.594	4	5	0.590	5	4	0.314
≥ 25	31	18	13		13	18		22	9	
ER										
Negative	19	9	10	0.181	6	13	0.157	13	6	0.587
Positive	21	14	7		11	10		14	7	
PR										
Negative	17	12	5	0.132	10	7	0.070	14	3	0.082
Positive	23	11	12		7	16		13	10	
PFS										
Yes	14	14	0	<0.001	14	0	<0.001	12	2	0.071
No	26	9	17		3	23		15	11	
OS										
Yes	13	13	0	<0.001	13	0	<0.001	11	2	0.105
No	27	10	17		4	23		16	11	

Abbreviations: D-dimer level before radition: D1; D-dimer level after radition: D2; Change in D-dimer level (D2 minus D1): ΔD; International Federation of Gynecology and Obstetrics: FIGO; G1: Well; G2: Moderate; G3: Poor; Myometrial invasion: MI; Body mass index: BMI; Estrogen receptor: ER; Progesterone receptor: PR; Progression-free survival: PFS; Overall survival: OS

### The relationship between D-dimer and IMRT response

We further assessed the relation between ΔD and IMRT response. The mean D2 level reduced by 0.74 mg/L compared to the D1 level in 14 patients with stable disease (SD, *P*= 0.032, Table 5). The mean D2 in 26 patients with progressive disease (PD) increased by 2.30 mg/L contradistinguished with the D1 (*P*= 0.003). The progression disease was significantly related to the increase of D-dimer after IMRT. Our data exhibited that the D-dimer level may be considered as a predictor for IMRT response in endometrial cancer patients.

**Table 5:** Differences in D-dimer levels in patients with PD and SD

Response (n=40)	D1(mg/L)	D2 (mg/L)	*P*
PD (n=26)	2.84±5.06	5.14±5.77	0.003
SD (n=14)	1.42±1.79	0.68±1.13	0.032
*P*	0.761	0.002	

Abbreviations: stable disease: SD; progressive disease: PD; D-dimer level before radition: D1; D-dimer level after radition: D2.

## DISCUSSION

Blood coagulation and fibrinolytic system play a significant role in tumor progression [12]. D-dimer serves as an indicator of fibrinolytic pathway activation, which is related to unfavorable prognosis in several cancers [7–10, 13, 14]. Our study is the study to investigate the correlation between D-dimer and survival in patients with endometrial cancer. Our data show that plasma D-dimer level is a predictor for the prognosis in endometrial cancer patients.

Furthermore, D-dimer level was also a prognosis marker in different FIGO stage subgroups. However, there was no statistical significance between the D-dimer level and stage IV group, which may be explained by the small sample size. The D-dimer concentration in FIGO stage II subgroup was lower compared to stage III and IV endometrial cancer. The level of D-dimer may be useful for disease staging. Patients should receive more treatments when a higher D-dimer levelis present. Moreover, endometrial cancer PD patients had an effectively higher D2 than SD patients. The D2 was higher in patients with PD while the D2 was lower in the patients with SD. Our data revealed that D-dimer might be important factors of patient prognosis and tumor response to IMRT.

The mechanism underlying the progression of endometrial cancer by D-dimer remains uncler. Nevertheless, the different D-dimer signaling pathways may play some roles. The abnormality of haemostasis and fibrinolysis during tumor development and progression illustrated the relationship between D-dimer levels and endometrial cancer progression [15–17]. Fbrinogen could be converted to fbrin by tumor cells and D-dimer is a degradation product of fibrinogen which increased in ongoing fbrinogen metabolism [18–20]. Enhanced expression of tissue factor, which is still expressed by tumor cells, could stimulate coagulation cascades and finally result in varieties of pathological processes, such as tumor progression, angiogenesis, and metastasis [15].

Nevertheless, some limitations should be paid attention in this study. This study is a retrospective and single-institution study. Another limitation is the small smaple size. The small cohort size may be insufficient statistical power to detect differences, and lack of generalisability to the wider population. The third limitation is that the data on venous thromboembolism (VTE) were not collected because D-dimer can predict VTE in cancer patients [21, 22]. It has been reported that both D-dimer and VTE are adverse prognostic indicator for cancer patients [23].

In summary, our data indicate that D-dimer levels before and after IMRT D-dimer is correlated with OS and PFS in endometrial cancer patients. Additionally, the D2 is associated with survival rate in patient with endometrial cancer. We demonstrated that D-dimer levels could be also used to assess prognosis and IMRT response in endometrial cancer patients. Further prospective research is needed to make sure these discoveries.

## MATERIALS AND METHODS

### Patients and clinical follow-up

The data of 40 patients with endometrial cancer patients in Shanghai Jiao Tong University Affiliated Sixth People's Hospital between 2006 and 2009 were collected in our study. The inclusion criteria of this study were the following: (1) patients with histological confirmed endometrial cancer based on the FIGO; (2) no previous treatment; (3) no previous others malignancies; and (4) none of the patients had taken in any anticoagulant drugs before enrollment. Ethical approval for the study protocols was obtained and informed consents were obtained from each patient. The mean follow-up period for these patients was 57 months (range: 9–84 months). All patients were followed up by determination of three monthly ultrasonography (US), computed tomography (CT) or magnetic resonance imaging (MRI) scan. The diagnosis of recurrence and metastases was based on two factors: histopathological findings of the tumor tissue in patients and on the characteristic appearance on US, CT and MRI.

### Data collection

We collected clinical data including age, BMI, FIGO stage, histologic grade, depths of MI, lymph node metastasis, KPS score, OS and PFS time and so on. D-dimer levels were detected before and after IMRT.

### Statistical analysis

Statistical analysis used the SPSS version 19.0 statistical software. Survival analysis was performed using the Kaplan–Meier method, and the log-rank test was used for analysis. Univariate and multivariate analyses were performed using Cox's proportional hazards model. The relationship between D-dimer levels and clinicopathological features were analyzed using χ2 test. The data are expressed as the mean±SEM, and *P* value < 0.05 was considered statistically.
